# Epidemiologic features of depression and anxiety among homeless adults with healthcare access problems in London, UK: descriptive cross-sectional analysis

**DOI:** 10.1192/bjo.2025.10956

**Published:** 2026-01-21

**Authors:** Sujit D. Rathod, PJ Annand, Paniz Hosseini, Andrew Guise, Lucy Platt

**Affiliations:** Department of Population Health, London School of Hygiene and Tropical Medicine, UK; Faculty of Social Sciences, University of Sheffield, UK; Department of Public Health, Environments and Society, London School of Hygiene and Tropical Medicine, UK; Department of Population Health Sciences, King’s College London, UK

**Keywords:** Depressive disorders, anxiety- or fear-related disorders, epidemiology, social deprivation, substance use disorders

## Abstract

**Background:**

In England, 354 000 people were homeless on a given night in 2024. It has long been recognised that the physical and mental health of people who are homeless is poorer than for those who are stably housed. There are few peer-reviewed studies to inform health- and social care around depression or anxiety for people who are homeless in this setting.

**Aims:**

To measure the symptoms of depression and anxiety among adults who are homeless and who have difficulty accessing healthcare, and to describe the distribution of symptoms across sociodemographic, health-related characteristics and indicators of social vulnerability.

**Method:**

We surveyed 311 adults between August and December 2021. We measured anxiety and depression symptoms using the four-item Patient Health Questionnaire (PHQ-4) score. We compared median PHQ-4 scores across strata of sociodemographic, social vulnerability and health-related characteristics, and tested for associations with the Kruskal–Wallis test.

**Results:**

The median PHQ-4 score was 7 out of 12, with 38% having scores warranting clinical attention. While PHQ-4 scores were consistently high across a range of socioeconomic, social vulnerability and health-related characteristics, they were positively associated with young age; food insecurity; recent and historic abuse; joint, bone or muscle problems; and marijuana use. The most common barrier to accessing healthcare related to transportation (60%).

**Conclusions:**

People who are homeless and have difficulty accessing healthcare have high levels of depression and anxiety symptoms. Our findings support further coordination between health- and social care sectors.

Approximately 354 000 people were homeless on a given night in England in 2024, including 322 000 in temporary accommodation and 3900 who were ‘sleeping rough’.^1^ The number of people who are homeless is increasing, due to growing challenges related to limited housing and widening economic insecurity.^2^ It has been recognised that the physical and mental health of people who are homeless is poorer than that of those who are stably housed.^3^ A global review of homeless people^4^ found that 47% report symptoms of depression, and between 11 and 26% have major depression or major depressive disorder; other reviews in high-income settings^5,6^ estimated prevalence figures of 11 and 12% for major depression. In the UK, a psychiatric survey of homeless people that was conducted in 1994^7^ estimated that the prevalence of depression or general anxiety disorder was 36%. In the Homeless Health Needs Audit report of 2022, over 60% of participants reported having depression or an anxiety disorder;^8^ in comparison, only 14% of adults living in private households had these conditions according to a national survey conducted in 1993, and 17% in 2014.^9^

## Medical and social implications of depression and anxiety among people who are homeless

Depression and anxiety can lead to disabling symptoms such as lack of motivation, energy and concentration, and chronic and excessive worry, which impair access to healthcare. Limited access to healthcare is exacerbated by high need, because those affected by depression or anxiety are more likely to experience the incidence, persistence and recurrence of other physical, mental and substance use conditions,^10^ and to attempt suicide.^11^ For homeless persons, access to health- and social care for the aforementioned conditions is hindered by stigma, discrimination and other intersecting domains of social exclusion, resulting in inequitable health outcomes: in England and Wales in 2021, the mean age at death was 45 and 43 years for homeless men and women, respectively,^12^ and homeless persons have standardised mortality ratios that are 2–5 times higher than those of the general population.^3^

## Epidemiologic evidence around depression and anxiety among people who are homeless

Evidence from systematic reviews on mental health among adults who are homeless^4–6^ is predominantly drawn from studies conducted in the USA, where the homeless population has distinct challenges including the high cost and poor availability of health- and social care. There is limited peer-reviewed evidence to inform health- and social care around depression or anxiety for homeless adults in the UK, where psychological care for depression and anxiety is available and free at the point of use but is not necessarily accessible (e.g. due to barriers with registration, opening hours, appointment booking systems, etc.). For evidence about depression, an association was found between dental problems and depression among 853 homeless adults in Scotland.^7^ Among patients of a specialist homelessness primary care centre in England, some 34% had depression and 23% had anxiety, which were associated with 1.8 and 2.3 times higher odds of non-fatal overdose, respectively.^13^ Evidence on anxiety is more limited: in one study of young people (16–24 years) in temporary supported accommodation, 19% had generalised anxiety disorder, which was associated with a 2.8-fold increase in emergency department use.^14^ A cluster analysis of 452 homeless adults^15^ found that those who had experience of being ‘very depressed or anxious’ clustered with those who had more complex and severe experiences of social exclusion and, furthermore, that symptoms tended to precede the exclusion.

With the population of homeless adults in England projected to increase, and access to healthcare – and, more specifically, to mental healthcare – remaining constrained, it is essential to characterise more fully the epidemiologic features of depression and anxiety, so that policy and research programmes can be prioritised appropriately.

## Method

### Setting/context

This study was undertaken in London, UK, where in 2024 an estimated 184 000 people were homeless, just over half of whom were adults.^1^ In England everyone is entitled to free primary and emergency healthcare through the National Health Service, regardless of residency status. For those with symptoms of depression or anxiety, self-referral to self-guided therapy is available while a GP can refer patients to talking therapy, opt for watchful waiting, prescribe medication or refer to mental health specialists. In addition to these services, people who are homeless can choose to register at a GP surgery that specialises in homeless healthcare, where available, and those staying in hostels may be able to access talking therapy through hostel-based counsellors. GPs and key workers can refer people with drug or alcohol dependency, or psychosis, directly to specialist mental healthcare, where comorbid depression or anxiety can be treated. While such entitlements are legally mandated, there are frequently a range of barriers to the availability and uptake of mental healthcare. These barriers include long wait lists; logistical and administrative challenges; past experiences of discrimination; lack of confidence; as well as low prioritisation of healthcare amid competing priorities of survival.

### Study procedures

This secondary analysis draws on data from an evaluation of a non-governmental organisation-delivered peer advocacy programme to improve access to healthcare, the methods for which have been reported in detail.^16^ Briefly, we recruited two cohorts from the same source population: adults experiencing homelessness in London and who had difficulty accessing healthcare. We recruited these cohorts concurrently between August and December 2021 and followed up for 12 months. The first cohort (‘clients’) were adults (≥25 years), homeless (e.g. sleeping rough, living in a hostel or shelter, in supported housing or with insecure private housing, etc.) in London, had difficulty accessing healthcare and were receiving support – whether new or ongoing – from the peer advocacy programme. Peer advocates – all of whom had lived experience of homelessness – recruited their clients into the cohort as part of their usual client engagement activities, with the understanding that consent or denial to the research would not affect the clients’ access to either the intervention or healthcare. For the second cohort (‘non-clients’), we recruited adults from hostels and day centres in London where the peer advocacy programme was not commissioned to work, and who either self-affirmed or had a key worker affirm their difficulty in accessing healthcare. A team of co-researchers – with experience of homelessness themselves or of working with homeless people – approached potential participants at these sites, explained the aims of the study and assessed them for eligibility. All participants who gave consent completed a 30 min structured questionnaire that could be either self- or interviewer-administered on a tablet device in either English or Polish. The questionnaire contained sections in three domains: (a) sociodemographic (e.g. age, gender, ethnicity); (b) social vulnerability (e.g. multiple-exclusion homelessness,^17^ food insecurity, history of incarceration and trauma); and (c) health (e.g. current conditions, barriers to care, drug and alcohol use and depression and anxiety symptoms).

### Study outcome

We used the 4-item PHQ-4,^18^ which uses a 4-point Likert scale for depression- and anxiety-related symptoms, with scores ranging from 0 (never) to 3 (nearly every day in the past 2 weeks). Because depression and anxiety are frequently comorbid and are increasingly considered to be a single psychological construct, and recognising the dimensional nature of mental health, most of our analyses used the sum of the 4 PHQ-4 items, a continuous measure (range 0–12) which, in this sample, had a Cronbach’s alpha of 0.83. We also created scoring categories of 0–5, 6–8 (‘yellow flag’) and 9–12 (‘red flag’), which were cut points suggested by Löwe et al^19^ as indicators for clinical attention.

### Other study measures

We measured age in years and grouped in 10-year intervals from 25. Participants could select one or more ethnic backgrounds (i.e. White, Asian, Black, Arab, Hispanic, other); we classified those who identified as White only, Black only and Asian only into their own categories, and the remainder (multiple ethnicity, other, decline to answer) into another category. We asked participants the age at which they first became homeless, subtracted that from their current age and created a category of <2 years to classify those who had recently become homeless, then divided the remaining participants into approximately equal-sized groups for 2–9, 10–24 and ≥25 years since first becoming homeless. For drinking frequency in the past 12 months, we re-categorised 9 frequency categories into 4: never, infrequent (up to a few times per year), frequent (a few times per month) and daily (5 or more days per week). For English literacy, we classified those who reported having below average ability in either reading or writing as having lower literacy. We included an 11-item tool to measure social vulnerability and exclusion characteristics for homeless persons.^17^ Participants who affirmed going hungry because they could not afford food in the past 12 months were classified as food insecure. We asked questions about violence using items drawn from the World Health Organization multi-country study on domestic violence,^20^ with recall periods of <6 and ≥6 months ago; those participants who reported ever having been pushed, shoved or slapped were classified as having physical abuse; ever having been belittled, humiliated or called racist names as verbal abuse; and ever having been touched, grabbed or forced sexually as sexual abuse. We asked participants about their non-prescription drug use in the past 12 months, and whether they used each of the drugs daily or less frequently. Those who had a PHQ-4 score of ≥9 and who daily used a substance (i.e. alcohol, cocaine, heroin, marijuana or spice) were classified as having a probable dual diagnosis.

### Ethics

The authors assert that all procedures contributing to this work comply with the ethical standards of the relevant national and institutional committees on human experimentation, and with the Helsinki Declaration of 1975 as revised in 2008. All procedures involving human subjects/patients were approved by Dulwich Research Ethics Committee (no. IRAS 271312) and the London School of Hygiene and Tropical Medicine (ref. 18021), both based in the UK. All participants provided written informed consent and were reimbursed £10 (cash or grocery voucher) for their time and offered a copy of *The Pavement* magazine, which includes a listing of services for homeless people in London.

### Statistical analysis

First we tabulated the sociodemographic, social vulnerability- and health-related characteristics of participants using proportions. To maintain confidentiality, we combined strata with <10 observations with other strata, if appropriate, or did not tabulate their findings. For large ‘choose all that apply’ question sets (e.g. ongoing health issues), we present the three to four most affirmed responses only. Second, we describe the sum of PHQ-4 scores using the median and interquartile range (IQR). We report the proportions with yellow and red flag PHQ-4 scores. Third, we report the median PHQ-4 score and IQR across each of the socioeconomic, social vulnerability- and health-related characteristics. Fourth, we consider evidence of association of these characteristics with PHQ-4. We could not use parametric tests such as the *t*-test or analysis of variance to test for associations because the PHQ-4 scores were skewed, and so we used the Kruskal–Wallis test, a non-parametric test, to compare the distribution of a continuous variable across two or more categories. As a descriptive analysis, our aim was to characterise, not to confirm, associations, and so we did not make adjustment for multiple testing. We used StataSE 18.0 for Windows (StataCorp, College Station, Texas, USA; www.stata.com) for this analysis (see supplementary file available at https://doi.org/10.1192/bjo.2025.10956). Fifth, we repeated steps 3 and 4 and tabulated results for the PHQ-4 symptom subscales for depression (PHQ2) and that for anxiety (GAD2). Finally, we conducted *post hoc*, pairwise comparisons of the categories from those measures with evidence of association in step 4, again with no adjustment for multiple testing.

## Results

We recruited 311 participants, of whom 153 were peer advocacy clients and 158 non-clients. The characteristics of the participants are shown in Table 1. Among all participants, 78.5% identified as male and 21.5% as female. Three participants identified as transgender and are not tabulated here. The median age was 48 years (IQR 40–57, not tabulated). The majority of participants were heterosexual (86.8%), identified as being of White ethnicity (65.3%), were UK citizens (78.1%), had completed only secondary school (52.4%) and had been sleeping in a hostel the previous night (70.1%). Notable proportions of participants had experienced verbal (49.8%) or physical (28.8%) abuse in the past 6 months, or sexual abuse in their lifetime (27.3%). The most common self-reported health problems were depression (75.2%), dental (57.6), joint, bone or muscle (46.0%), addiction (43.4%) and respiratory (41.8%), and 60.5% reported having problems with transportation to access healthcare. Using suggested cut points to guide clinical practice,^19^ 38.2% had red flag PHQ-4 scores of 9–12 and a further 25.0% had yellow flag scores of 6–8; 7 participants (2.2%) did not respond to at least 1 item of PHQ-4 and were not included in further analyses.

**Table 1:** Sociodemographic, social exclusion and health-related characteristics, and their association with PHQ-4 scores, among homeless health peer advocacy evaluation cohort participants, London, UK, 2020–2021

Measure and stratum	Total (%)	PHQ-4 median score (IQR)	Kruskal–Wallis *P*-value
Total sample	311 (100)	7 (4–10)	0.95
Non-client cohort	158 (50.80)	7 (4–10)	
Client cohort	153 (49.20)	7 (4–10)	
Sociodemographic characteristics			
Gender			0.76
Male	244 (78.5)	7 (4–10)	
Female	67 (21.5)	7 (4–10)	
Age category, years			0.01
25–34	40 (12.9)	8 (5–11)	
35–44	78 (25.1)	8 (5–10)	
45–54	98 (31.5)	7 (4–10)	
55–64	78 (25.1)	6 (3–9)	
≥65	17 (5.4)	1 (1–8)	
Sexual orientation			0.51
Heterosexual	270 (86.8)	7 (4–10)	
Gay, lesbian	11 (3.5)	7 (4–8)	
Bisexual	11 (3.5)	9 (5–12)	
Other, don’t know	19 (6.1)	9 (6–10)	
Ethnicity			0.96
White only	203 (65.3)	7 (4–10)	
Asian/British Asian only	41 (13.2)	7.5 (5–9)	
Black/Black British only	27 (8.7)	7 (4–12)	
Other, multiple, decline to answer	40 (12.9)	8 (4–10)	
Citizenship			0.29
Other	68 (21.9)	6.5 (4–10)	
UK	243 (78.1)	7 (4–10)	
Education completed			0.98
Less than secondary	40 (13.0)	7 (4–10)	
Secondary	161 (52.4)	7 (4–10)	
More than secondary	106 (34.5)	7 (4–10)	
English language literacy			0.41
Better than average reading and writing	224 (72.0)	7 (4–10)	
Below average reading or writing	87 (28.0)	7 (3–10)	
Social vulnerability characteristics			
Years since first became homeless category			0.04
0–1	29 (9.3)	8 (6–9)	
2–9	102 (32.8)	7 (3–10)	
10–24	92 (29.6)	8 (5–11)	
≥25	88 (28.3)	6 (3–10)	
Sleeping location, last night			0.49
Slept rough/public location	24 (7.7)	8 (5.5–12)	
Hostel	218 (70.1)	7 (4–10)	
Own tenancy	32 (10.3)	6.5 (4–10)	
Other	37 (11.9)	9 (4–11)	
Sofa-surfed, ever			0.92
Yes	238 (76.5)	7 (4–10)	
No	73 (23.5)	7 (4–10)	
Lived in hostel or refuge, ever			0.17
Yes	275 (88.4)	7.5 (4–10)	
No	36 (11.6)	6.5 (3–10)	
Slept rough or in public location, ever			0.74
Yes	264 (84.9)	7 (4–10)	
No	47 (15.1)	8 (4–10)	
Applied to council as homeless, ever			0.62
Yes	219 (70.4)	7 (4–10)	
No	92 (29.6)	7 (4–10)	
Local authority care as a child, ever			0.40
Yes	75 (24.1)	6.5 (4–9)	
No	236 (75.9)	7 (4–10)	
Begged, ever			0.45
Yes	128 (41.2)	7 (5–10)	
No	183 (58.8)	7 (4–10)	
Shop-lifted, ever			0.29
Yes	139 (44.7)	8 (4–10)	
No	172 (55.3)	7 (4–10)	
Daily binge-drinking, ever			0.53
Yes	190 (61.1)	7 (4–10)	
No	118 (37.9)	7 (3–10)	
Street-drinking, ever			0.73
Yes	163 (52.4)	7 (4–10)	
No	145 (46.6)	8 (4–10)	
Sold sex, ever			0.57
Yes	30 (9.6)	6.5 (4–9)	
No	281 (90.4)	7 (4–10)	
Incarcerated, ever			0.80
Yes	155 (49.8)	7 (4–10)	
No	156 (50.2)	7.5 (4–10)	
Injected drugs, ever			0.25
Yes	90 (28.4)	8 (5–10)	
No	219 (70.4)	7 (4–10)	
Arrested, detained or charged by police, past 6 months			0.99
Yes	49 (15.8)	7 (4–10)	
No	258 (83.0)	7 (4–10)	
Told to move from public space by police, past 6 months			0.14
Yes	86 (27.6)	8 (5–11)	
No	221 (71.1)	7 (4–10)	
Food insecure, past 12 months			0.01
No	121 (38.9)	5 (2–8)	
Yes	187 (60.1)	8 (5–12)	
Verbal abuse			0.01
Never	77 (24.8)	5 (1–8)	
Last ≥6 months ago	76 (24.4)	7 (4–10)	
Within past 6 months	152 (48.9)	8 (5–12)	
Physical abuse			0.01
Never	107 (34.4)	6 (3–9)	
Last ≥6 months ago	110 (35.4)	8 (4–10)	
Within past 6 months	88 (28.3)	8 (5–11)	
Sexual abuse, ever			0.04
No	226 (72.7)	7 (4–10)	
Yes	85 (27.3)	8 (5–11)	
Health characteristics			
Depression or anxiety problems, current			0.01
Yes	234 (75.2)	8 (5–11)	
No	77 (24.8)	3 (1–6)	
Dental problems, current			0.35
Yes	179 (57.6)	7 (4–10)	
No	132 (42.4)	7 (4–10)	
Joint, bone or muscle problems, current			0.03
Yes	143 (46.0)	8 (5–11)	
No	168 (54.0)	7 (3.5–10)	
Addiction problems, current			0.07
Yes	135 (43.4)	8 (5–10)	
No	176 (56.6)	7 (4–10)	
Respiratory (e.g. obstructive airway disease, bronchitis, emphysema, asthma) problems, current			0.26
Yes	130 (41.8)	7 (5–10)	
No	181 (58.2)	7 (4–10)	
Has problems with transport to access healthcare			0.01
Yes	188 (60.5)	8 (5–11)	
No	123 (39.5)	5 (3–9)	
Has uncertainty about healthcare place or provider			0.01
Yes	185 (59.5)	8 (5–11)	
No	126 (40.5)	6 (3–9)	
Has difficulty getting a healthcare appointment			0.01
Yes	161 (51.8)	8 (5–11)	
No	150 (48.2)	6 (3–9)	
Crack or cocaine used, 12 months			0.29
No	186 (59.8)	7 (3–10)	
Yes, less than daily	97 (31.2)	8 (5–10)	
Yes, daily	28 (9.0)	8 (4.5–11.5)	
Heroin used, 12 months			0.32
No	220 (70.7)	7 (4–10)	
Yes, less than daily	72 (23.1)	7 (5–10)	
Yes, daily	19 (6.1)	9 (6–11)	
Marijuana used, 12 months			0.01
No	190 (61.1)	7 (3.5–9.5)	
Yes, less than daily	81 (26.0)	8 (4–10)	
Yes, daily	40 (12.9)	9.5 (6–12)	
Daily substance (alcohol, crack, cocaine, heroin, marijuana, spice) use, 12 months			0.02
No	245 (78.8)	7 (4–10)	
Yes	66 (21.2)	8 (6–12)	
Alcohol consumption, 12 months			0.98
Never	73 (23.5)	7 (4–10)	
Infrequent (up to a few days per year)	75 (24.1)	7 (4–10)	
Frequent (up to a few days per month)	75 (24.1)	7 (4–10)	
Daily	81 (26.0)	7 (4–10)	
PHQ-4 score category			
0–5, ‘green flag’	112 (36.8)		
6–8, ‘yellow flag’	76 (25.0)		
9–12, ‘red flag’	116 (38.2)		
Probable dual diagnosis (red flag + daily substance use)			
No	253 (81.3)		
Yes	54 (17.4)		

PHQ-4, 4-item Patient Health Questionnaire; IQR, interquartile range.

The overall median PHQ-4 score was 7, with IQR of 4–10. Both male and female participants had a median PHQ-4 score of 7, with IQR of 4–10, and there was no evidence of a difference in these distributions (Kruskal–Wallis, *P* = 0.76). There was evidence of an association of PHQ-4 score with age, in that scores were highest among younger participants (median 8) and lower among older participants (median 1). Scores were highest among those whose first episode of homelessness occurred in the past year (median 8), and lowest among those who were first homeless over 25 years ago (median 6). People who reported food insecurity had higher scores (median 8) than those who were food secure (median 5). Recent or historic experiences of physical, verbal and sexual abuse were all associated with higher PHQ-4 scores (all *P* < 0.05). Those who had joint, bone or muscle problems had higher scores (median 8) than those who did not (median 7). The three most common barriers to healthcare were all associated with higher scores: for example, participants who identified transportation as a barrier had a median score of 8, compared with a median of 5 for those who did not. Those who used marijuana daily had higher scores (median 9.5) than those who did not (median 7). There was no evidence of difference in PHQ-4 scores across crack/cocaine, heroin or alcohol consumption categories (all *P* > 0.29). The results for analyses of the PHQ2 depression symptom subscale are reported in Supplementary Table 2, for the GAD2 anxiety symptom subscale in Supplementary Table 3 and *post hoc* pairwise testing of PHQ-4 in Supplementary Table 4.

## Discussion

### Key findings

Among homeless adults who reported issues accessing healthcare, depression and anxiety symptoms were high, with a median PHQ-4 score of 7 out of 12, and 38% having red flag scores warranting clinical attention. Awareness of these conditions by name was also high: over 75% of participants affirmed having ongoing problems with ‘depression or anxiety’. While PHQ-4 scores were consistently high across participant characteristics, there was evidence that these were higher among younger people, those using marijuana daily, those who had transportation problems and those who had joint, bone or muscle problems. In addition, almost 2 in 3 participants had experienced hunger in the past 12 months, 1 in 2 had experienced verbal abuse in the past 6 months and 1 in 3 had experienced sexual abuse in their lifetime.

### Burden of symptoms and coverage of care

The depression and anxiety symptom levels observed in this sample are far higher than those found in previous meta-analyses of homeless populations,^4–6^ and are probably an artefact of a study inclusion criterion: self-reported difficulty in accessing healthcare. For this population, reducing the burden of these conditions will require consideration of both their incidence and duration. Because insecure housing is evidenced as an incidence risk factor for a range of social and medical conditions,^21^ action to improve housing security must remain a priority. To reduce duration, we need to develop more accessible forms of psychological support. For example, models of peer support interventions have been developed and demonstrated as being effective in improving mental health;^22^ one peer-delivered model for substance use counselling among people who are homeless is currently being evaluated in the UK.^23^

### Associations with depression and anxiety symptoms

We observed higher PHQ-4 scores among younger age groups. This trend is consistent with one study from Taiwan^24^ and the opposite of another from the USA.^25^ We also observed higher scores among those who first became homeless recently. Two studies,^26,27^ both from San Francisco, USA, found that recent homelessness was associated with depression and anxiety; in further analysis, these two measures were positively correlated. It is plausible that younger people had higher PHQ-4 scores because they had more recently became homeless, although it is also plausible that older participants had lower PHQ-4 scores due to survivor bias. Our findings support efforts to address the drivers of housing insecurity.

The lack of association here between sexual orientation and both depression and anxiety is contrary to findings from the general population in the UK,^28^ and among homeless people in the USA.^26,29,30^ Overall, 11% of our sample identified as gay, lesbian, bisexual or other – substantially higher than the 3% estimated from national population surveys in the UK^28^ but the same as that reported in the 2022 Homeless Health Needs Audit.^8^ The disproportionate representation of sexual minorities in the homeless population is well characterised^31^ and, given the lack of research on mental health among sexual minorities within the homeless population, we recommend further qualitative research and larger quantitative studies to consider whether there are protective factors for sexual minorities that are present in the UK and absent in other settings.

Around 6 in 10 of our participants had experienced hunger due to lack of funds in the past 12 months, reinforcing the 2022 Homeless Health Need Audit in England,^8^ which found that only 18% of respondents had an average of 3 meals per day. Participants who had experienced hunger had higher PHQ-4 scores than those who had not. Similar findings have been reported from Canada and the USA,^27,32,33^ with the cohort study from Palar et al^33^ finding that severe food insecurity precedes an increase in depression severity. These findings further support the need for a strengthening of social care services, so that social work teams can devote adequate time to help their clients navigate disparate services (i.e. for nutrition, housing, physical health and mental health), and can sensitise the relevant service providers to the intersecting vulnerabilities faced by their clients.

The use of drugs and alcohol to self-medicate against symptoms of mental distress has been well characterised in the general population, and has been reported from studies of homeless persons in the USA and France.^34,35^ In this study, we did not detect any associations among crack, heroin or alcohol use with PHQ-4 scores, although we did with marijuana. Building on the findings of Lowe et al,^36^ our findings support further research on whether cannabis use exacerbates physical health issues and depression symptoms, and whether the cognitive effects of cannabis might be related to some of the specific barriers to accessing healthcare (e.g. relating to transportation) reported by our participants. We observed that 1 in 5 participants was using a substance daily and that 17% had a probable dual diagnosis (e.g. high PHQ-4 score and daily substance use), evidencing a confluence of homelessness, substance use and mental distress. This finding should motivate further coordination between social and medical providers.

The high prevalence of verbal, physical and sexual abuse is consistent with review evidence^3^ that estimates between 27 and 52% of homeless persons had experienced physical or sexual assault in the previous year. As evidenced both in the UK and internationally,^15,34,37–39^ abuse is associated with depression and anxiety, and with other domains of social exclusion. Homeless service providers need to ensure that health and social services are informed by an understanding of, and sensitivity around, experience of violence and the role that this trauma might play in the lives and behaviours of service users. This understanding might include the development of organisational cultures that foster an understanding of how experiences of adversity may shape behaviour, and improvement in staff training around how to recognise and respond to the signs of trauma. Improving access to specialist trauma services for people experiencing homelessness is also key. Inclusion of those with lived experience of homelessness services as staff and volunteers could also facilitate systemic change, to better recognise and meet the needs of clients who face historic and ongoing abuse. Furthermore, these findings reinforce the need for integration of housing and mental health support for survivors of abuse.

Consistent with a study of people who are homeless in Los Angeles, USA,^40^ we found high levels of physical joint, bone or muscle problems associated with higher PHQ-4 scores. While somatic symptoms are common features of depression, these physical problems can sometimes be independent comorbidities. This finding highlights the need for integrated approaches to care, such that presentation with these common physical complaints is routinely accompanied by investigation into depression and anxiety and vice versa, and that clinical providers coordinate their care. We also note the high concordance of PHQ-4 scores with self-reported problems of depression and anxiety. The fact that participants with symptoms recognised their underlying conditions evidences an encouraging level of mental health literacy, upon which further interventions to improve access to care can build.

All participants in this analysis had difficulty accessing healthcare according to the eligibility criteria of the study; the most common barriers included problems with transportation, uncertainty about location and inability to get an appointment. These barriers were associated with higher PHQ-4 scores. We recommend that policy-makers consider removal of structural barriers to services, including those around accessing public transport, and of barriers to referrals within and between medical and social care sectors.

### Strengths and limitations

This is the first descriptive analysis, using validated tools, of the distribution of depression and anxiety symptoms among any homeless population in the UK. Given emerging findings about peer support for the health of homeless people, we believe that our engagement with peers and co-researchers with lived experience of homelessness means that many of our participants were people who would otherwise be hesitant to participate in a research study, and were more likely to provide complete and accurate responses. We believe that this engagement resulted in less selection bias and less response bias. The high levels of affirmation to question items that are considered stigmatising (e.g. on abuse, substance use and mental health) evidences a level of trust to disclose to our co-researchers who, like peer advocates, have lived experience of homelessness. Our measure of depression and anxiety symptoms was PHQ-4, a subset of items drawn from PHQ9 and GAD7,^18^ which are well characterised and widely used to screen for depression and anxiety, respectively. For analysis, we used PHQ-4 as a continuous measure, which is consistent with the dimensional approach to mental health and avoids the clinical and measurement issues inherent in using a cut-off score to diagnose a mental health condition. There are several limitations of this analysis that must be noted. First, it is not possible to make causal interpretations for the associations detected here; our data are cross-sectional and temporality is not always clear. Second, no sampling frame exists for homeless people in London, nor could we recruit from all hostels and day centres in this city because we relied on cooperation from site managers. Furthermore, given the focus of the primary study from which this secondary analysis was drawn – about people who have difficulty accessing healthcare in London – our sample may be systematically different to the broader population of people who are homeless. And third, because some strata in this analysis were relatively small, our statistical power to detect modest variations in PHQ-4 scores was limited. While there were over 40 statistical tests run in the main analysis (Table 1), we did not make adjustments for multiple comparisons. Due to the risk of type 1 error (false positive) associated with multiple testing, readers should treat our findings as hypothesis-generating rather than confirmatory.

## Supporting information

Rathod et al. supplementary material 1 Rathod et al. supplementary material

Rathod et al. supplementary material 2 Rathod et al. supplementary material

## Data Availability

The data that support the findings of this study are available from L.P. (lucy.platt@lshtm.ac.uk), upon reasonable request.

## Supplementary material

The supplementary material is available online at https://doi.org/10.1192/bjo.2025.10956

## References

[1] Shelter. Homelessness in England 2024. Shelter, 2024 (https://assets.ctfassets.net/6sxvmndnpn0s/5KyqSpsRrcgwqgXukTVoys/084db0ad0172b16a912a996c8aaa49f4/2024_Shelter_Big_Number_Report.pdf [cited 10 Feb 2025]).

[2] Public Health England. Homelessness: Applying All Our Health. Public Health England, 2019 (https://www.gov.uk/government/publications/homelessness-applying-all-our-health/homelessness-applying-all-our-health) [cited 3 Jun 2022]).

[3] Fazel S , Geddes JR , Kushel M. The health of homeless people in high-income countries: descriptive epidemiology, health consequences, and clinical and policy recommendations. Lancet 2014; 384: 1529–40. 25390578 10.1016/S0140-6736(14)61132-6 PMC4520328

[4] Ayano G , Belete A , Duko B , Tsegay L , Dachew BA. Systematic review and meta-analysis of the prevalence of depressive symptoms, dysthymia and major depressive disorders among homeless people. BMJ Open 2021; 11: e040061. 10.1136/bmjopen-2020-040061 PMC7907847 33622940

[5] Fazel S , Khosla V , Doll H , Geddes J. The prevalence of mental disorders among the homeless in Western countries: systematic review and meta-regression analysis. PLOS Med 2008; 5: e225. 19053169 10.1371/journal.pmed.0050225 PMC2592351

[6] Gutwinski S , Schreiter S , Deutscher K , Fazel S. The prevalence of mental disorders among homeless people in high-income countries: an updated systematic review and meta-regression analysis. PLOS Med 2021; 18: e1003750. 34424908 10.1371/journal.pmed.1003750 PMC8423293

[7] Gill B , Meltzer H , Hinds K. The prevalence of psychiatric morbidity among homeless adults. Int Rev Psychiatry 2003; 15: 134–40. 12745321 10.1080/0954026021000046056

[8] Hertzberg D , Boobis S . *The Unhealthy State of Homelessness 2022: Findings from the Homeless Health Needs Audit*. Homeless Link, 2022 (https://homeless.org.uk/documents/754/Homeless_Health_Needs_Audit_Report.pdf [cited 7 Jan 2026]).

[9] McManus S , Bebbington P , Jenkins R , Brugha T. *Mental Health and Wellbeing in England*. UK Department of Health and Social Care, 2014 (https://files.digital.nhs.uk/pdf/s/5/adult_psychiatric_study_executive_summary_web.pdf [cited 7 Jan 2026]).

[10] Grenard JL , Munjas BA , Adams JL , Suttorp M , Maglione M , McGlynn EA , et al. Depression and medication adherence in the treatment of chronic diseases in the United States: a meta-analysis. J Gen Intern Med 2011; 26: 1175–82. 21533823 10.1007/s11606-011-1704-y PMC3181287

[11] Lee KH , Jun JS , Kim YJ , Roh S , Moon SS , Bukonda N , et al. Mental health, substance abuse, and suicide among homeless adults. J Evid Inf Soc Work 2017; 14: 229–42. 28678621 10.1080/23761407.2017.1316221

[12] Office for National Statistics. Deaths of Homeless People in England and Wales: 2021 Registrations. ONS, 2022 (https://www.ons.gov.uk/peoplepopulationandcommunity/birthsdeathsandmarriages/deaths/bulletins/deathsofhomelesspeopleinenglandandwales/2021registrations [cited 10 Feb 2025]).

[13] Anderson J , Kurmi O , Lowrie R , Araf A , Paudyal V. Patterns, circumstances and risk factors associated with non-fatal substance overdose in a cohort of homeless population: an observational study. Int J Clin Pharm 2025; 47: 107–18. 39560880 10.1007/s11096-024-01812-z PMC11748478

[14] Hodgson KJ , Shelton KH , van den BMBM. Mental health problems in young people with experiences of homelessness and the relationship with health service use: a follow-up study. Evid Based Ment Health 2014; 17: 76–80. 25043432 10.1136/eb-2014-101810 PMC13404263

[15] Fitzpatrick S , Bramley G , Johnsen S. Pathways into multiple exclusion homelessness in seven uk cities. Urban Stud 2013; 50: 148–68.

[16] Rathod SD , Guise A , Annand P , Hosseini P , Williamson E , Miners A , et al. Peer advocacy and access to healthcare for people who are homeless in London, UK: a mixed method impact, economic and process evaluation protocol. BMJ Open 2021; 11: e050717. 10.1136/bmjopen-2021-050717 PMC8212404 34140346

[17] Fitzpatrick S , Johnsen S , White M. Multiple exclusion homelessness in the UK: key patterns and intersections. Soc Policy Soc 2011; 10: 501–12.

[18] Kroenke K , Spitzer RL , Williams JBW , Löwe B. An ultra-brief screening scale for anxiety and depression: the PHQ–4. Psychosomatics 2009; 50: 613–21. 19996233 10.1176/appi.psy.50.6.613

[19] Löwe B , Wahl I , Rose M , Spitzer C , Glaesmer H , Wingenfeld K , et al. 4-item measure of depression and anxiety: validation and standardization of the Patient Health Questionnaire-4 (PHQ-4) in the general population. J Affect Disord 2010; 122: 86–95. 19616305 10.1016/j.jad.2009.06.019

[20] Garcia-Moreno C , Jansen HAFM , Ellsberg M , Heise L , Watts C. *WHO Multi-Country Study on Women’s Health and Domestic Violence Against Women: Initial Results on Prevalence, Health Outcomes and Women’s Responses*. World Health Organization, 2005 (https://apps.who.int/iris/handle/10665/43309 [cited 7 Jan 2026]).

[21] Gallent N. Whose Housing Crisis? Assets and Homes in a Changing Economy. Policy Press, 2019.

[22] Smit D , Miguel C , Vrijsen JN , Groeneweg B , Spijker J , Cuijpers P. The effectiveness of peer support for individuals with mental illness: systematic review and meta-analysis. Psychol Med 2023; 53: 5332–41. 36066104 10.1017/S0033291722002422 PMC10476060

[23] Parkes T , Matheson C , Carver H , Foster R , Budd J , Liddell D , et al. A peer-delivered intervention to reduce harm and improve the well-being of homeless people with problem substance use: the SHARPS feasibility mixed-methods study. Health Technol Assess 2022; 26: 1–128. 10.3310/WVVL4786 PMC8899911 35212621

[24] La Gory M , Ritchey FJ , Mullis J. Depression among the homeless. J Health Soc Behav 1990; 31: 87–102. 2313079

[25] Wang LY , Lin LP , Chen YC , Wang TW , Lin JD. Correlates of depressive symptoms among middle-aged and older homeless adults using the 9-item Patient Health Questionnaire. Int J Environ Res Public Health 2020; 17: 4754. 32630635 10.3390/ijerph17134754 PMC7370065

[26] Flentje A , Shumway M , Wong LH , Riley ED. Psychiatric risk in unstably housed sexual minority women: relationship between sexual and racial minority status and human immunodeficiency virus and psychiatric diagnoses. Womens Health Issues 2017; 27: 294–301. 28108194 10.1016/j.whi.2016.12.005 PMC5435529

[27] Riley ED , Dilworth SE , Satre DD , Silverberg MJ , Neilands TB , Mangurian C , et al. Factors associated with symptoms of depression and anxiety among women experiencing homelessness and unstable housing during the COVID-19 pandemic. JAMA Netw Open 2021; 4: e2117035. 34259851 10.1001/jamanetworkopen.2021.17035 PMC8281009

[28] Semlyen J , King M , Varney J , Hagger-Johnson G. Sexual orientation and symptoms of common mental disorder or low wellbeing: combined meta-analysis of 12 UK population health surveys. BMC Psychiatry 2016; 16: 67. 27009565 10.1186/s12888-016-0767-z PMC4806482

[29] Rohde P , Noell J , Ochs L , Seeley JR. Depression, suicidal ideation and STD-related risk in homeless older adolescents. J Adolesc 2001; 24: 447–60. 11549325 10.1006/jado.2001.0382

[30] Schick V , Witte L , Misedah L , Benedict W , Falk K , Brown C , et al. Exploring differences in the lives and well-being of sexual and gender minority adults experiencing homelessness relative to their cisgender heterosexual counterparts. Health Equity 2019; 3: 68–72. 31032470 10.1089/heq.2018.0068 PMC6484342

[31] Fraser B , Pierse N , Chisholm E , Cook H. LGBTIQ+ homelessness: a review of the literature. Int J Environ Res Public Health 2019; 16: 2677. 31357432 10.3390/ijerph16152677 PMC6695950

[32] Lachaud J , Mejia-Lancheros C , Wang R , Wiens K , Nisenbaum R , Stergiopoulos V , et al. Mental and substance use disorders and food insecurity among homeless adults participating in the At Home/Chez Soi study. PLOS One 2020; 15: e0232001. 32324795 10.1371/journal.pone.0232001 PMC7179857

[33] Palar K , Kushel M , Frongillo EA , Riley ED , Grede N , Bangsberg D , et al. Food insecurity is longitudinally associated with depressive symptoms among homeless and marginally-housed individuals living with HIV. AIDS Behav 2015; 19: 1527–34. 25351185 10.1007/s10461-014-0922-9 PMC4414669

[34] Fond G , Tinland A , Boucekine M , Girard V , Loubière S , Boyer L , et al. Improving the treatment and remission of major depression in homeless people with severe mental illness: the multicentric French Housing First (FHF) program. Prog Neuropsychopharmacol Biol Psychiatry 2020; 99: 109877. 31987919 10.1016/j.pnpbp.2020.109877

[35] Berg J , Nyamathi A , Christiani A , Morisky D , Leake B. Predictors of screening results for depressive symptoms among homeless adults in Los Angeles with latent tuberculosis. Res Nurs Health 2005; 28: 220–9. 15884031 10.1002/nur.20074 PMC3109748

[36] Lowe DJE , Sasiadek JD , Coles AS , George TP. Cannabis and mental illness: a review. Eur Arch Psychiatry Clin Neurosci 2019; 269: 107–20. 30564886 10.1007/s00406-018-0970-7 PMC6397076

[37] Tsai AC , Weiser SD , Dilworth SE , Shumway M , Riley ED. Violent victimization, mental health, and service utilization outcomes in a cohort of homeless and unstably housed women living with or at risk of becoming infected with HIV. Am J Epidemiol 2015; 181: 817–26. 25834138 10.1093/aje/kwu350 PMC4423526

[38] Padgett DK , Struening EL. Victimization and traumatic injuries among the homeless: associations with alcohol, drug, and mental problems. Am J Orthopsychiatry 1992; 62: 525–34. 1443061 10.1037/h0079369

[39] Larney S , Conroy E , Mills KL , Burns L , Teesson M. Factors associated with violent victimisation among homeless adults in Sydney, Australia. Aust N Z J Public Health 2009; 33: 347–51. 19689595 10.1111/j.1753-6405.2009.00406.x

[40] Nyamathi A , Branson C , Idemundia F , Reback C , Shoptaw S , Marfisee M , et al. Correlates of depressed mood among young stimulant-using homeless gay and bisexual men. Issues Ment Health Nurs 2012; 33: 641–9. 23017039 10.3109/01612840.2012.691605 PMC3624023

