# Supplementary material for: Epidemiologic features of depression and anxiety among homeless adults with healthcare access problems in London, UK: descriptive cross-sectional analysis
Source: BJPsych Open. 2026 Jan 21;12(1):e46. doi: 10.1192/bjo.2025.10956 (PMC12835710; doi:10.1192/bjo.2025.10956)
Supplement: Rathod et al. supplementary material 1 — Rathod et al. supplementary material [file S2056472425109563sup001.docx]

Supplementary Table 2. Sociodemographic, social exclusion, and health related characteristics, and association with PHQ2 depression score among homeless health peer advocacy clients and non-clients, London, United Kingdom, 2020-21.

| **Measure and Stratum** | | **PHQ2 median score (IQR)** | **Kruskal-Wallis P-value** |
| --- | --- | --- | --- |
| Total sample | | 4 (2-6) |  |
| Client cohort | | 4 (2-6) | 0.82 |
| Non-client cohort | | 4 (2-5.5) |  |
| Sociodemographic characteristics | | | |
| Gender | |  | 0.62 |
|  | Male | 4 (2-5) |  |
|  | Female | 3 (2-6) |  |
| Age category, years | |  | 0.01 |
|  | 25-34 | 4 (2-6) |  |
|  | 35-44 | 4 (2-5) |  |
|  | 45-54 | 4 (2-6) |  |
|  | 55-64 | 3 (1-4) |  |
|  | >=65 | 1 (0-4) |  |
| Sexual orientation | |  | 0.78 |
|  | Heterosexual | 4 (2-6) |  |
|  | Gay, lesbian | 3 (2-4) |  |
|  | Bisexual | 4 (2-6) |  |
|  | Other, don’t know | 3.5 (3-5) |  |
| Ethnicity | |  | 0.99 |
|  | White only | 4 (2-6) |  |
|  | Black/Black British only | 4 (2-6) |  |
|  | Asian/British Asian only | 3 (2-5) |  |
|  | Other, multiple, decline to answer | 4 (2-6) |  |
| Citizenship | |  | 0.55 |
|  | Other | 4 (2-5) |  |
|  | United Kingdom | 4 (2-6) |  |
| Education completed | |  | 0.54 |
|  | Less than secondary | 3 (1.5-4.5) |  |
|  | Secondary | 4 (2-6) |  |
|  | More than secondary | 4 (2-5) |  |
| English literacy | |  | 0.80 |
|  | Better than average reading and writing | 4 (2-5) |  |
|  | Below average reading or writing | 4 (1-6) |  |
| Social vulnerability characteristics | | | |
| Years since first became homeless category | |  | 0.05 |
|  | 0-1 | 4 (2-6) |  |
|  | 2-9 | 3 (2-4.5) |  |
|  | 10-24 | 4 (2-6) |  |
|  | >=25 | 3.5 (1-5) |  |
| Sleep location, last night | |  |  |
|  | Slept rough / public location | 4 (3-6 | 0.41 |
|  | Hostel | 4 (2-5) |  |
|  | Own tenancy | 3 (2-6) |  |
|  | Other | 3 (2-6) |  |
| Sofa surfed, ever | |  | 0.81 |
|  | Yes | 4 (2-5) |  |
|  | No | 4 (2-6) |  |
| Lived in hostel or refuge, ever | |  | 0.50 |
|  | Yes | 4 (2-6) |  |
|  | No | 3 (2-6) |  |
| Slept rough or in public location, ever | |  | 0.93 |
|  | Yes | 4 (2-5.5) |  |
|  | No | 4 (2-6) |  |
| Applied to council as homeless, ever | |  | 0.51 |
|  | Yes | 4 (2-6) |  |
|  | No | 4 (2-5) |  |
| Local authority care as a child, ever | |  | 0.56 |
|  | Yes | 3 (2-5) |  |
|  | No | 4 (2-6) |  |
| Begged, ever | |  | 0.37 |
|  | Yes | 4 (2-6) |  |
|  | No | 3 (2-5) |  |
| Shoplifted, ever | |  | 0.30 |
|  | Yes | 4 (2-6) |  |
|  | No | 3 (2-6) |  |
| Daily binge drinking, ever | |  | 0.71 |
|  | Yes | 4 (2-5) |  |
|  | No | 4 (2-6) |  |
| Street drinking, ever | |  | 0.89 |
|  | Yes | 4 (2-5) |  |
|  | No | 4 (2-6) |  |
| Sold sex, ever | |  | 0.37 |
|  | Yes | 3.5 (2-4) |  |
|  | No | 4 (2-6) |  |
| Incarcerated, ever | |  | 0.90 |
|  | Yes | 4 (2-5) |  |
|  | No | 4 (2-6) |  |
| Injected drugs, ever | |  | 0.16 |
|  | Yes | 4 (2-6) |  |
|  | No | 3 (2-6) |  |
| Arrested, detained, or charged by police, 6 mo | |  | 0.95 |
|  | Yes | 3 (2-6) |  |
|  | No | 4 (2-5) |  |
| Told to move from public space by police, 6 mo | |  | 0.78 |
|  | Yes | 3 (2-6) |  |
|  | No | 4 (2-5) |  |
| Food insecure, 12 mo | |  | 0.01 |
|  | No | 3 (1-4) |  |
|  | Yes | 4 (2-6) |  |
| Verbal abuse | |  | 0.01 |
|  | Never | 3 (1-4) |  |
|  | Last >=6 months ago | 4 (2-5) |  |
|  | Within past 6 months | 4 (2-6) |  |
| Physical abuse | |  | 0.02 |
|  | Never | 3 (1-4) |  |
|  | Last >=6 months ago | 4 (2-5) |  |
|  | Within past 6 months | 4 (2-6) |  |
| Sexual abuse, ever | |  | 0.24 |
|  | No | 3 (2-5) |  |
|  | Yes | 4 (2-6) |  |
|  | | | |
| Dental problems, current | |  | 0.78 |
|  | Yes | 4 (2-6) |  |
|  | No | 4 (2-5) |  |
| Joint, bone, or muscle problems, current | |  | 0.03 |
|  | Yes | 4 (2-6) |  |
|  | No | 3 (2-5) |  |
| Addiction problems, current | |  | 0.06 |
|  | Yes | 4 (2-6) |  |
|  | No | 3 (2-5) |  |
| Respiratory (e.g. obstructive airway disease, bronchitis, emphysema, and asthma) problems, current | |  | 0.28 |
|  | Yes | 4 (2-6) |  |
|  | No | 4 (2-5) |  |
| Depression or anxiety problems, current | |  | 0.01 |
|  | Yes | 4 (3-6) |  |
|  | No | 2 (0-3) |  |
| Has problems with transport to get health care | |  | 0.01 |
|  | Yes | 4 (2-6) |  |
|  | No | 3 (1-4) |  |
| Has uncertainty about health care place or provider | |  | 0.01 |
|  | Yes | 4 (2-6) |  |
|  | No | 3 (2-4) |  |
| Has difficulty getting health care appointment | |  | 0.01 |
|  | Yes | 4 (2-6) |  |
|  | No | 3 (1-4) |  |
| Crack or cocaine used, 12 mo | |  | 0.48 |
|  | No | 3 (2-6) |  |
|  | Yes, less than daily | 4 (2-5) |  |
|  | Yes, daily | 4 (2-6) |  |
| Heroin used, 12 mo | |  | 0.53 |
|  | No | 3 (2-6) |  |
|  | Yes, less than daily | 4 (2-5) |  |
|  | Yes, daily | 4 (3-6) |  |
| Marijuana used, 12 mo | |  | 0.01 |
|  | No | 3 (2-5) |  |
|  | Yes, less than daily | 4 (2-5) |  |
|  | Yes, daily | 4 (2.5-6) |  |
| Daily substance (alcohol, crack, cocaine, heroin, marijuana, spice) use, 12 mo | |  | 0.09 |
|  | No | 3 (2-5) |  |
|  | Yes | 4 (2-6) |  |
| Alcohol consumption, 12 mo | |  | 0.98 |
|  | Never | 3 (2-6) |  |
|  | Infrequent (up to a few days a year) | 4 (2-6) |  |
|  | Frequent (up to a few days a month) | 4 (2-5) |  |
|  | Daily | 4 (2-5) |  |

IQR, Interquartile range; PHQ2, 2-item Patient’s Health Questionnaire

Supplementary Table 3. Sociodemographic, social exclusion, and health related characteristics, and association with GAD2 anxiety score among homeless health peer advocacy clients and non-clients, London, United Kingdom, 2020-21.

| **Measure and Stratum** | | **GAD2 median score (IQR)** | **Kruskal-Wallis P-value** |
| --- | --- | --- | --- |
| Total sample | | 4 (2-6) |  |
| Client cohort | | 4 (2-6) | 0.68 |
| Non-client cohort | | 4 (2-6) |  |
| Sociodemographic characteristics | | | |
| Gender | |  | 0.96 |
|  | Male | 4 (2-6) |  |
|  | Female | 3.5 (2-6) |  |
| Age category, years | |  | 0.01 |
|  | 25-34 | 4 (2.5-6) |  |
|  | 35-44 | 4 (2-6) |  |
|  | 45-54 | 4 (2-6) |  |
|  | 55-64 | 3 (1-5) |  |
|  | >=65 | 1 (0-4) |  |
| Sexual orientation | |  | 0.39 |
|  | Heterosexual | 4 (2-6) |  |
|  | Gay, lesbian | 3 (2-6) |  |
|  | Bisexual | 3.5 (3-6) |  |
|  | Other, don’t know | 5 (3-6) |  |
| Ethnicity | |  | 0.82 |
|  | White only | 4 (2-6) |  |
|  | Black/Black British only | 4 (0-6) |  |
|  | Asian/British Asian only | 4 (3-5) |  |
|  | Other, multiple, decline to answer | 4 (2-6) |  |
| Citizenship | |  | 0.20 |
|  | Other | 3 (1-5.5) |  |
|  | United Kingdom | 4 (2-6) |  |
| Education completed | |  | 0.69 |
|  | Less than secondary | 4 (2-6) |  |
|  | Secondary | 4 (2-6) |  |
|  | More than secondary | 4 (2-6) |  |
| English literacy | |  | 0.21 |
|  | Better than average reading and writing | 4 (2-6) |  |
|  | Below average reading or writing | 3 (1-5) |  |
| Social vulnerability characteristics | | | |
| Years since first became homeless category | |  | 0.05 |
|  | 0-1 | 4 (2-5) |  |
|  | 2-9 | 4 (2-6) |  |
|  | 10-24 | 4 (3-6) |  |
|  | >=25 | 3 (1-6) |  |
| Sleep location, last night | |  | 0.66 |
|  | Slept rough / public location | 4 (2.5-6) |  |
|  | Hostel | 4 (2-6) |  |
|  | Own tenancy | 4 (2-6) |  |
|  | Other | 4 (2-6) |  |
| Sofa surfed, ever | |  | 0.89 |
|  | Yes | 4 (2-6) |  |
|  | No | 4 (2-6) |  |
| Lived in hostel or refuge, ever | |  | 0.15 |
|  | Yes | 4 (2-6) |  |
|  | No | 2.5 (1-6) |  |
| Slept rough or in public location, ever | |  | 0.37 |
|  | Yes | 4 (2-6) |  |
|  | No | 4 (2-6) |  |
| Applied to council as homeless, ever | |  | 0.79 |
|  | Yes | 4 (2-6) |  |
|  | No | 4 (2-6) |  |
| Local authority care as a child, ever | |  | 0.41 |
|  | Yes | 3 (2-6) |  |
|  | No | 4 (2-6) |  |
| Begged, ever | |  | 0.52 |
|  | Yes | 4 (2-6) |  |
|  | No | 4 (2-6) |  |
| Shoplifted, ever | |  | 0.37 |
|  | Yes | 4 (2-6) |  |
|  | No | 4 (2-6) |  |
| Daily binge drinking, ever | |  | 0.43 |
|  | Yes | 4 (2-6) |  |
|  | No | 4 (1-6) |  |
| Street drinking, ever | |  | 0.71 |
|  | Yes | 4 (2-6) |  |
|  | No | 4 (2-6) |  |
| Sold sex, ever | |  | 0.99 |
|  | Yes | 3.5 (2-5) |  |
|  | No | 4 (2-6) |  |
| Incarcerated, ever | |  | 0.75 |
|  | Yes | 3.5 (2-6) |  |
|  | No | 4 (2-6) |  |
| Injected drugs, ever | |  | 0.43 |
|  | Yes | 4 (2-6) |  |
|  | No | 4 (2-6) |  |
| Arrested, detained, or charged by police, 6 mo | |  | 0.96 |
|  | Yes | 4 (2-6) |  |
|  | No | 4 (2-6) |  |
| Told to move from public space by police, 6 mo | |  | 0.03 |
|  | Yes | 4 (2-6) |  |
|  | No | 3 (2-6) |  |
| Food insecure, 12 mo | |  | 0.01 |
|  | No | 2 (1-4) |  |
|  | Yes | 4 (2-6) |  |
| Verbal abuse | |  | 0.01 |
|  | Never | 2.5 (0-4) |  |
|  | Last >=6 months ago | 3 (2-5) |  |
|  | Within past 6 months | 4.5 (2-6) |  |
| Physical abuse | |  | 0.01 |
|  | Never | 3 (1-4) |  |
|  | Last >=6 months ago | 4 (2-6) |  |
|  | Within past 6 months | 4 (2-6) |  |
| Sexual abuse, ever | |  | 0.01 |
|  | No | 4 (2-5) |  |
|  | Yes | 4 (2-6) |  |
|  | | | |
| Dental problems, current | |  | 0.19 |
|  | Yes | 4 (2-6) |  |
|  | No | 4 (2-5) |  |
| Joint, bone, or muscle problems, current | |  | 0.06 |
|  | Yes | 4 (2-6) |  |
|  | No | 4 (2-6) |  |
| Addiction problems, current | |  | 0.15 |
|  | Yes | 4 (2-6) |  |
|  | No | 4 (1-6) |  |
| Respiratory (e.g. obstructive airway disease, bronchitis, emphysema, and asthma) problems, current | |  | 0.33 |
|  | Yes | 4 (2-6) |  |
|  | No | 4 (2-6) |  |
| Depression or anxiety problems, current | |  | 0.01 |
|  | Yes | 4 (2-6) |  |
|  | No | 1 (0-3) |  |
| Has problems with transport to get health care | |  | 0.01 |
|  | Yes | 4 (2-6) |  |
|  | No | 2 (1-5) |  |
| Has uncertainty about health care place or provider | |  | 0.01 |
|  | Yes | 4 (2-6) |  |
|  | No | 3 (1-5) |  |
| Has difficulty getting health care appointment | |  | 0.01 |
|  | Yes | 4 (2-6) |  |
|  | No | 3 (1-5) |  |
| Crack or cocaine used, 12 mo | |  | 0.21 |
|  | No | 3.5 (1.5-6) |  |
|  | Yes, less than daily | 4 (2-6) |  |
|  | Yes, daily | 4 (2-6) |  |
| Heroin used, 12 mo | |  | 0.26 |
|  | No | 4 (2-6) |  |
|  | Yes, less than daily | 4 (2-6) |  |
|  | Yes, daily | 5 (3-6) |  |
| Marijuana used, 12 mo | |  | 0.04 |
|  | No | 3 (2-6) |  |
|  | Yes, less than daily | 4 (2-6) |  |
|  | Yes, daily | 4.5 (3-6) |  |
| Daily substance (alcohol, crack, cocaine, heroin, marijuana, spice) use, 12 mo | |  | 0.01 |
|  | No | 3 (2-6) |  |
|  | Yes | 4 (2-6) |  |
| Alcohol consumption, 12 mo | |  | 0.94 |
|  | Never | 4 (2-5) |  |
|  | Infrequent (up to a few days a year) | 4 (2-6) |  |
|  | Frequent (up to a few days a month) | 4 (2-6) |  |
|  | Daily | 4 (2-6) |  |

IQR, Interquartile range; GAD2, 2-item Generalised Anxiety Disorder screening tool

Supplementary Table 4. P-values for pairwise comparison of PHQ4 score distributions among homeless health peer advocacy clients and non-clients, London, United Kingdom, 2020-21.

| Age category, years | | Median (IQR) | vs 25-34 | vs 35-44 | vs 45-54 | vs 55-64 |
| --- | --- | --- | --- | --- | --- | --- |
|  | 25-34 | 4 (2.5-6) |  |  |  |  |
|  | 35-44 | 4 (2-6) | 0.31 |  |  |  |
|  | 45-54 | 4 (2-6) | 0.18 | 0.31 |  |  |
|  | 55-64 | 3 (1-5) | 0.01 | 0.01 | 0.01 |  |
|  | >=65 | 1 (0-4) | 0.01 | 0.01 | 0.01 | 0.08 |
| Years since first became homeless category | | Median (IQR) | vs 0-1 | vs 2-9 | vs 10-24 |  |
|  | 0-1 | 8 (6-9) |  |  |  |  |
|  | 2-9 | 7 (3-10) | 0.35 |  |  |  |
|  | 10-24 | 8 (5-11) | 0.18 | 0.09 |  |  |
|  | >=25 | 6 (3-10) | 0.06 | 0.10 | 0.010 |  |
| Verbal abuse | | Median (IQR) | vs Never | vs Last >=6 mo ago |  |  |
|  | Never | 5 (1-8) |  |  |  |  |
|  | Last >=6 months ago | 7 (4-10) | 0.13 |  |  |  |
|  | Within past 6 months | 8 (5-12) | 0.01 | 0.14 |  |  |
| Physical abuse | | Median (IQR) | vs Never | vs Last >=6 mo ago |  |  |
|  | Never | 6 (3-9) |  |  |  |  |
|  | Last >=6 months ago | 8 (4-10) | 0.01 |  |  |  |
|  | Within past 6 months | 8 (5-11) | 0.01 | 0.22 |  |  |
| Marijuana used, 12 mo | | Median (IQR) | vs No | vs Yes, less than daily |  |  |
|  | No | 7 (3.5-9.5) |  |  |  |  |
|  | Yes, less than daily | 8 (4-10) | 0.13 |  |  |  |
|  | Yes, daily | 9.5 (6-12) | 0.01 | 0.03 |  |  |
