# Supplementary material for: Epidemiologic features of depression and anxiety among homeless adults with healthcare access problems in London, UK: descriptive cross-sectional analysis
Source: BJPsych Open. 2026 Jan 21;12(1):e46. doi: 10.1192/bjo.2025.10956 (PMC12835710; doi:10.1192/bjo.2025.10956)
Supplement: Rathod et al. supplementary material 2 — Rathod et al. supplementary material [file S2056472425109563sup002.docx]

********************

*** Housekeeping ***

********************

use "questionnaire.dta", clear

keep study_id eligible consent arm years phq1 phq2 gad1 gad2 phq2sum gad2sum phq4sum phq4flag ///

age_5c age gender trans sexorient4c eth4c citizen_uk edu_3c lowlit ///

sleep4c meh_* sexsell jailed arrestsix prisonsix movesix food verbal3c physical3c sexual ///

health_depression health_teeth health_joint health_drug breath barriers_* helped ///

cracoke3c heroin3c weed3c dailydrug drinkfreq4c dualdx ///

oppre oppredna inpre edpre oppost oppostdna edpost inpost

***********************

*** PHQ4 score dist ***

***********************

alpha phq? gad?, d

* Sociodems, inclusion and health vars

forval arm=0/2 {

foreach y of varlist phq4sum phq2sum gad2sum {

sum `y', detail

foreach var of varlist gender age_5c sexorient4c eth4c citizen_uk ///

edu_3c lowlit years4c sleep4c meh_* sexsell jailed arrestsix movesix food ///

verbal3c physical3c sexual ///

health_teeth health_joint health_drug breath health_dep ///

barriers_trans barriers_unsure barriers_appoint ///

cracoke3c heroin3c weed3c dailydrug drinkfreq4c dualdx {

tab `var' if arm==`arm', m

table `var' if arm==`arm', stat(n `y') stat(p50 `y') stat(p25 `y') stat(p75 `y')

kwallis `y' if `var'!=99 & `var'!=88 & arm==`arm', by(`var')

* dunntest `y' if `var'!=99 & `var'!=88, by(`var')

}

}

}

* Post hoc tests on significant categorical vars in the main analysis

foreach var of varlist age_5c years4c verbal3c physical3c weed3c {

dunntest phq4sum if `var'!=88 & `var'!=99, by(`var') ma(none)

}
